# STALARD: Selective Target Amplification for Low-Abundance RNA Detection

**DOI:** 10.1186/s13007-025-01443-z

**Published:** 2025-09-29

**Authors:** Daesong Jeong, Chulmin Park, Ilha Lee

**Affiliations:** 1 Laboratory of Plant Developmental Genetics, School of Biological Sciences, Seoul National University, Seoul, 08826 Korea; 2 Research Center for Plant Plasticity, School of Biological Sciences, Seoul National University, Seoul, 08826 Korea

**Keywords:** Low-abundance RNA, Reverse transcription-quantitative real-time PCR, Isoform quantification, Selective transcript enrichment

## Abstract

**Background:**

Accurate quantification of RNA isoforms is critical for understanding gene regulation. However, conventional reverse transcription-quantitative real-time PCR (RT-qPCR) has limited sensitivity for low-abundance transcript isoforms, as quantification cycle (Cq) values above 30 are often considered unreliable. While transcriptome-wide analyses can address this limitation, they require costly deep sequencing and complex bioinformatics. Moreover, isoform-specific qPCR is often confounded by differential primer efficiency when comparing similar transcripts.

**Results:**

To overcome the sensitivity and amplification bias limitations of conventional RT-qPCR for detecting known low-abundance and alternatively spliced transcripts, we developed STALARD (*Selective Target Amplification for Low-Abundance RNA Detection*), a rapid (< 2 h) and targeted two-step RT-PCR method using standard laboratory reagents. STALARD selectively amplifies polyadenylated transcripts sharing a known 5′-end sequence, enabling efficient quantification of low-abundance isoforms. When applied to *Arabidopsis thaliana*, STALARD successfully amplified the low-abundance *VIN3* transcript to reliably quantifiable levels. Amplification of *FLM*, *MAF2*, *EIN4*, and *ATX2* isoforms by STALARD reflected known splicing changes during vernalization, including cases where conventional RT-qPCR failed to detect relevant isoforms. STALARD also enabled consistent quantification of the extremely low-abundance antisense transcript *COOLAIR*, resolving inconsistencies reported in previous studies. In combination with nanopore sequencing, STALARD further revealed novel *COOLAIR* polyadenylation sites not captured by existing annotations.

**Conclusion:**

STALARD provides a sensitive, simple, and accessible method for isoform-level quantification of low-abundance transcripts that share a known 5’-end sequence. Its compatibility with both qPCR and long-read sequencing makes it a versatile tool for analyzing transcript variants and identifying previously uncharacterized 3′-end structures, provided that isoform-specific 5′-end sequences are known in advance.

**Supplementary Information:**

The online version contains supplementary material available at 10.1186/s13007-025-01443-z.

## Background

Plants dynamically regulate gene expression during development and in response to environmental cues [1]. Therefore, accurate quantification of transcripts is essential for understanding these complex processes. In eukaryotes, alternative splicing (AS) greatly increases transcriptome complexity by enabling the production of multiple messenger RNA (mRNA) isoforms with distinct functions from a single gene [2–4]. These isoforms often differ in stability and translation efficiency, adding an important layer of post-transcriptional regulation [5].

Alternative splicing is widespread in plants, affecting more than 60% of genes, and plays a critical role in response to environmental stimuli and developmental signals [6–11]. For example, *FLOWERING LOCUS M* (*FLM*) and *MADS AFFECTING FLOWERING 2* (*MAF2*) regulate flowering time in response to temperature via AS. The *FLM-β* isoform accumulates at low temperatures to delay flowering, while *FLM-δ* increases at high temperatures to promote flowering. Similarly, the *MAF2var1* isoform is highly expressed under vernalization conditions to repress flowering [12–15]. Additionally, *COOLAIR*, an antisense transcript from the *FLOWERING LOCUS C* (*FLC*) locus, exists in multiple isoforms partly due to AS [16–19]. Proximal polyadenylation of *COOLAIR*, mediated by autonomous pathway (AP) proteins involved in flowering, has been proposed to contribute to the epigenetic suppression of *FLC* [20–26].

Precise quantification of transcript isoforms is essential to fully understand the regulatory consequences of AS, yet it remains technically challenging. Many isoforms are expressed at low levels, resulting in quantification cycle (Cq) values that exceed the lower limit of quantification (LLOQ) for reverse transcription-quantitative real-time PCR (RT-qPCR). According to the Minimum Information for Publication of Quantitative Real-Time PCR Experiments (MIQE) guidelines, Cq values above 30–35 are often considered unreliable due to poor reproducibility [27–32]. Furthermore, conventional isoform-specific qPCR requires distinct primer pairs that introduce amplification bias due to differences in primer efficiency, which is a challenge, especially causing problems for low-abundance transcripts.

Digital PCR (dPCR) improves sensitivity but requires specialized reagents and instrumentation [33]. Similarly, transcriptome-wide analyses offer a comprehensive view of RNA populations but often fail to accurately quantify low-abundance isoforms without deep sequencing and complex bioinformatic analysis [34]. These limitations underscore the need for targeted, sensitive, cost-effective, and accessible methods for accurate isoform quantification.

To address this need, we developed STALARD (*Selective Target Amplification for Low-Abundance RNA Detection*), a targeted pre-amplification strategy designed to overcome both low transcript abundance and primer-induced bias. This method combines conventional reverse transcription and targeted PCR, similar in concept to the Switching Mechanism At 5’-End of RNA Template (SMART) approach [35], but STALARD is optimized for quantifying polyadenylated isoforms that share a defined 5’-end sequence. While STALARD is not intended for discovering novel transcription start sites, it can detect both known and previously unannotated isoforms that differ in splicing or 3′-end usage, making it a valuable tool for profiling transcript diversity.

**Fig. 1:**
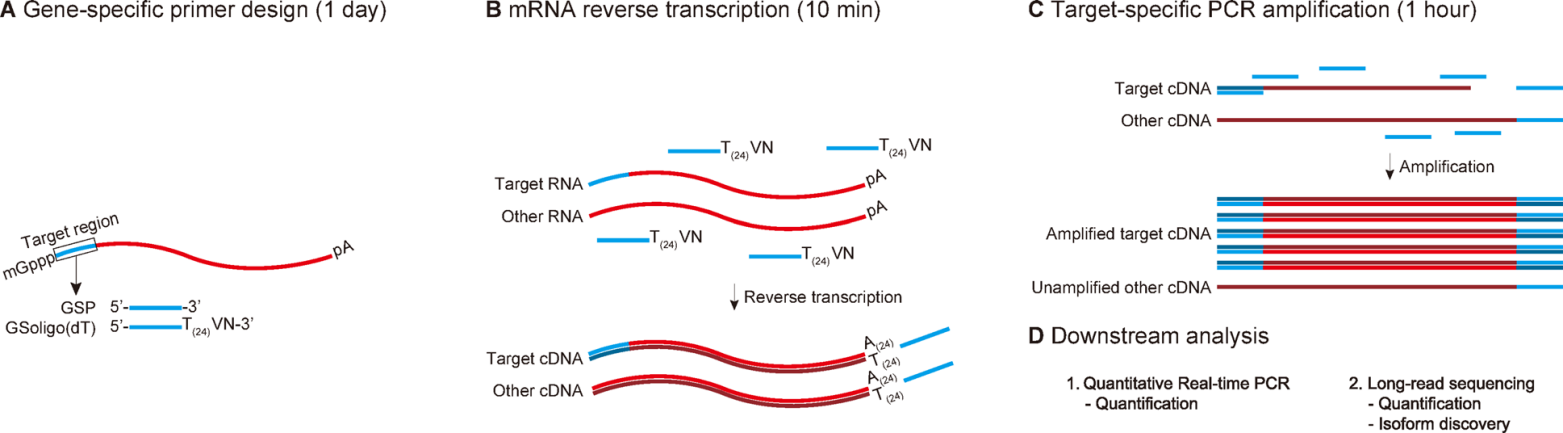
Schematic overview of the STALARD workflow. **A**. Primer design using GSoligo(dT) (a gene-specific primer-tailed oligo(dT)_24_VN) for reverse transcription and a gene-specific primer (GSP) for PCR. **B**. Reverse transcription of mRNA using GSoligo(dT) under standard RT conditions. **(C)** Target-specific PCR amplification using the first-strand cDNA generated in panel B and the GSP. **(D)** Downstream analysis of the PCR products from panel C

STALARD employs a two-step, target-specific pre-amplification prior to quantification (Fig. 1). First, reverse transcription is performed using an oligo(dT) primer tailed at its 5′-end with a gene-specific sequence that matches the 5′ end of the target RNA (with T substituted for U). This gene-specific adapter is incorporated into the resulting cDNA. In the second step, limited-cycle PCR (< 12 cycles) is performed using only this gene-specific primer, which now anneals to both ends of the cDNA. This approach specifically amplifies the target transcript without requiring a separate reverse primer, thereby minimizing amplification bias caused by primer selection and reducing nonspecific amplification.

We validated STALARD using RNA extracted from *Arabidopsis thaliana* seedlings subjected to vernalization. We first targeted *VERNALIZATION INSENSITIVE 3* (*VIN3*), which is expressed at low levels under non-vernalized conditions (requiring Cq > 30 for detection). We then tested *FLM*, *MAF2*, *ETHYLENE INSENSITIVE 4* (*EIN4*), and *ARABIDOPSIS TRITHORAX 2* (*ATX2*) to assess whether STALARD could efficiently amplify multiple isoforms while preserving relative abundance. These genes exhibit distinct AS patterns and varying expression levels under vernalized and non-vernalized conditions [36]. Finally, we applied STALARD to *COOLAIR*, an extremely low-abundance antisense non-coding transcript of *FLC*, as a stringent test of its ability to detect and quantify individual transcript isoforms. Our results demonstrate that STALARD accurately captures AS patterns regardless of transcript abundance. STALARD provides a sensitive, simple, and accessible solution for targeted transcript analysis, overcoming limitations of existing methods and enabling accurate quantification of low-abundance isoforms.

## Materials and methods

### Plant materials and growth conditions

*Arabidopsis thaliana* Columbia-0 (Col-0) and Col-*FRI*^*Sf2*^ (*FRI*-Col) were used as wild-type controls. The *FRI*-Col, *ld-1*, *fca-9*, and *fve-4* have been previously described [37–40]. The mutants *fld-3* (*SALK_075401*), *fpa-7* (*SALK_138449*), and *fy-2* (*SAIL_657_D04*) were used to detect and quantify *COOLAIR* isoforms.

Seeds were sown on half-strength Murashige and Skoog (MS) medium (Duchefa, Haarlem, Netherlands) containing 1% sucrose and 1% plant agar (Duchefa), then stratified at 4 °C for 3 days to promote uniform germination. Seedlings were grown at 22 °C under either long-day (LD; 16 h light/8 hr dark) or short-day (SD; 8 h light/16 hr dark) conditions under cool white fluorescent lights (120 µmol·m^− 2^·s^− 1^ for LD, 100 µmol·m^− 2^·s^− 1^ for SD). For vernalization treatment, *FRI-*Col seedlings were first grown at 22 °C for 14 days, then transferred to 4 °C growth chamber under SD conditions for 20 days. Non-vernalized (NV) plants were grown at 22 °C for 16 days. To quantify *COOLAIR* transcript quantification, Col-0, *fca-9*, *fld-3*, *fpa-7*, *fve-4*, *fy-2*, and *ld-1* plants were grown at 22 °C for 7 days under LD conditions.

### RNA extraction and cDNA synthesis

Total RNA was extracted from 100 mg of seedlings grown on MS agar plates using Nucleozol (MACHEREY-NAGEL, Duren, Germany) following the manufacturer’s instructions. First-strand cDNA was synthesized from 1 µg of total RNA using the HiScript IV 1st Strand cDNA Synthesis Kit (Vazyme, Nanjing, China), according to the manufacturer’s protocol, and used directly for quantification.

### STALARD

Two types of primers were used: a gene-specific primer (GSP), and a GSP-tailed oligo(dT)_24_VN primer (GSoligo(dT); where V = adenine (A), guanine (G), or cytosine (C) and N = any bases). GSPs were designed to match the 5′-end sequences of the target RNA (with thymine replacing uracil), enabling specific annealing to both ends of the cDNA and facilitating efficient amplification. Primer3 software was used to select GSPs with a melting temperature (Tm) of 62 °C, GC content of 40–60%, and no predicted hairpin or self-dimer structures.

First-strand cDNA was synthesized from 1 µg of total RNA using the HiScript IV 1st Strand cDNA Synthesis Kit (Vazyme, Nanjing, China) and 1 µL of 50 µM GSoligo(dT) primer. The resulting cDNA carried the GSP sequence at its 5’ end. PCR amplification was then performed using 1 µL of 10 µM GSP and SeqAmp DNA polymerase (Takara, Shiga, Japan) in a 50 µL reaction. Thermal cycling was as follows: initial denaturation at 95 °C for 1 min; 9–18 cycles of 98 °C for 10 s (denaturation), 62 °C for 30 s (annealing), and 68 °C for 1 min per kb (extension); and a final extension at 72 °C for 10 min.

PCR products were purified using AMPure XP beads (Beckman Coulter, Brea, CA, USA) at a 1.0:0.7 (product: beads) ratio and eluted in 300 µL of RNase-free water for qPCR or in 10 µL for nanopore sequencing library preparation.

### Quantitative real-time PCR (qPCR) and analysis

qPCR was performed using BioFACT™ 2X Real-Time PCR Master Mix^ⓐ^, containing EvaGreen™ in the mixture, with Low Rox reference dye (Biofact, Daejeon, Republic of Korea), according to the manufacturer’s instructions, on a CFX96 Real-Time PCR system (Bio-Rad). Relative transcript levels were calculated using the 2^−ΔCq^ method, and fold-changes were determined using the 2^−ΔΔCq^ method [27–29].

### Nanopore sequencing and analysis

Amplicon libraries were prepared using the Native Barcoding Kit 24 V14 (Oxford Nanopore, Oxford, UK) according to the manufacturer’s instructions. Sequencing was performed on a MinION Mk1B using an R10.4.1 flow cell. Raw reads were trimmed using Porechop v0.2.4 [41], and the trimmed reads were mapped to the ARAPORT11 reference genome using minimap2 v2.28 [42]. Transcript counts were obtained with Bambu v3.8.3 to calculate the ratio of proximal-to-distal *COOLAIR* isoforms [43]. To assess polyadenylation site usage, all outputs were merged and mapped. Usage of each site was calculated directly from the mapped read coverage.

## Results

### Selective target amplification for low-abundance RNA detection (STALARD)

Quantifying transcripts with Cq values above 30 is often unreliable due to poor reproducibility, as this range approaches the lower limit of quantification (LLOQ) for conventional RT-qPCR methods [30–32]. In *Arabidopsis thaliana*, several genes with critical developmental roles, such as *VIN3*, are expressed at levels near this threshold. Therefore, reliable quantification of such low-abundance transcripts and their isoforms is necessary to understand plant development.

To address this challenge, we developed STALARD (Selective Target Amplification for Low-Abundance RNA Detection), a method that enables targeted pre-amplification of low-abundance transcripts prior to quantification. STALARD is designed for use with polyadenylated RNAs that share a known 5′-end sequence, which is used for primer design.

As outlined in Fig. 1, STALARD uses a gene-specific (GSP)-tailed oligo(dT) primer during reverse transcription and a single gene-specific primer during limited-cycle PCR, allowing precise and selective amplification of target RNAs with minimal off-target effects. The resulting pre-amplified product can then be used in downstream quantification steps such as qPCR or nanopore sequencing.

We validated STALARD’s performance using *Arabidopsis* transcripts with low expression levels (Fig. 1A–D). The method reliably enriched target products, reduced background amplification, and completed the entire workflow, from RNA to amplified product, in under two hours. STALARD is compatible with both qPCR and sequencing-based quantification, providing a sensitive and flexible tool for transcript isoform analysis.

### Evaluating STALARD efficiency and optimizing PCR cycle number

**Fig. 2:**
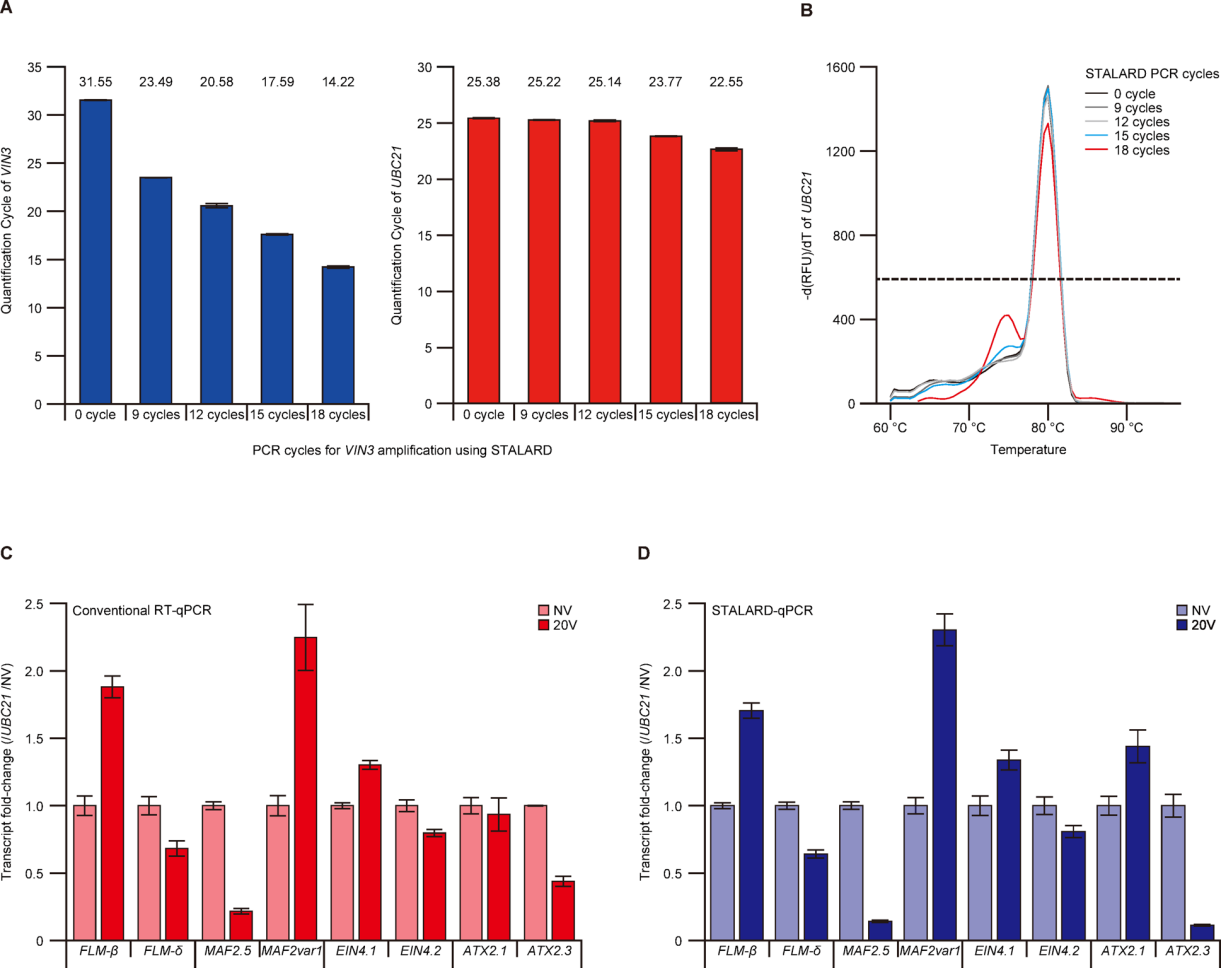
Validation of STALARD pre-amplification. **A**. Quantification of *VIN3* and *UBC21* transcripts by qPCR following 0, 9, 12, 15, or 18 cycles of STALARD pre-amplification. Data represent the mean ± SD from three technical replicates. **B.** Melt peak analysis of *UBC21* STALARD-qPCR products. **C**. Fold changes in alternatively spliced isoforms of four genes, *FLM*,* MAF2*,* EIN4*, and *ATX2*, under NV and 20 V conditions, measured using conventional RT-qPCR. Transcript levels are normalized to NV and shown as mean ± standard error of the mean (SEM) from three biological replicates. **D**. Same as in panel C, but measured using STALARD-qPCR

To assess the amplification efficiency and potential bias of STALARD, we tested the method using the low-abundance *VIN3* transcript under non-vernalized (NV) conditions. We performed 0, 9, 12, 15, or 18 cycles of STALARD PCR and quantified *VIN3* and a reference gene, *UBIQUITIN-CONJUGATING ENZYME 21* (*UBC21*), using qPCR. Without STALARD amplification (0 cycle), the Cq value of *VIN3* averaged 31.55, which falls within the LLOQ range (Fig. 2A). As the number of STALARD PCR cycles increased, *VIN3* Cq values decreased linearly, indicating efficient amplification (Fig. 2A). In contrast, *UBC21* Cq values remained stable up to 12 cycles, but began to decrease significantly beyond 12 cycles, likely due to non-specific amplification products, as indicated by a Tm of 74 °C (Fig. 2A, B). These results suggest that STALARD efficiently amplifies target transcripts while minimizing off-target effects. Based on these findings, we set STALARD amplification at 12 cycles for all subsequent experiments to minimize amplification bias.

### Reliable isoform quantification across a wide range of transcript abundances using STALARD

To evaluate whether STALARD preserves the relative abundance of transcript isoforms, we tested four genes with well-characterized alternative splicing (AS) patterns during vernalization: *FLM*,* MAF2*,* EIN4*, and *ATX2*. Based on previous transcriptome data [36], these genes span a broad range of expression levels: *FLM* and *MAF2* are relatively abundant (TPM > 30), while *EIN4* and *ATX2* are expressed at much lower levels (TPM < 10). This expression range provided a suitable testbed for assessing whether STALARD can amplify target isoforms without distorting their relative abundance.

We first compared the quantification range of conventional RT-qPCR versus STALARD-qPCR targeting *FLM*,* MAF2*,* EIN4*, and *ATX2* isoforms. STALARD increased relative target levels (normalized to *UBC21*) from a range of 0.0001–1.5 (conventional RT-qPCR) to 0.1–35,000 (STALARD-qPCR), bringing low-abundance targets into a quantifiable range (Supplemental Fig. 1A, B).

We then analyzed AS patterns and isoform abundance in NV and 20-day vernalized (20 V) samples. As expected from previous studies, *FLM*, *MAF2*, and *EIN4* exhibited consistent AS patterns in both conventional RT-qPCR and STALARD-qPCR. In contrast, AS patterns of *ATX2* were only recapitulated after STALARD pre-amplification. Conventional RT-qPCR failed to resolve these isoforms, likely due to Cq values exceeding 30 and approaching the LLOQ (Fig. 2C, D).

Isoform fold changes between NV and 20 V conditions followed similar trends. For *FLM*, *MAF2*, and *EIN4*, fold changes matched previous transcriptomic profiles [36] regardless of pre-amplification. However, for *ATX2*, fold changes were only consistent with prior transcriptomic data when STALARD was applied (Fig. 2C, D). These results confirm that STALARD is suitable for quantifying previously characterized isoforms across a wide dynamic range, provided that isoform-specific 5′ sequences are known.

### Tackling an enigmatic target: resolving *COOLAIR* isoform quantification

**Fig. 3:**
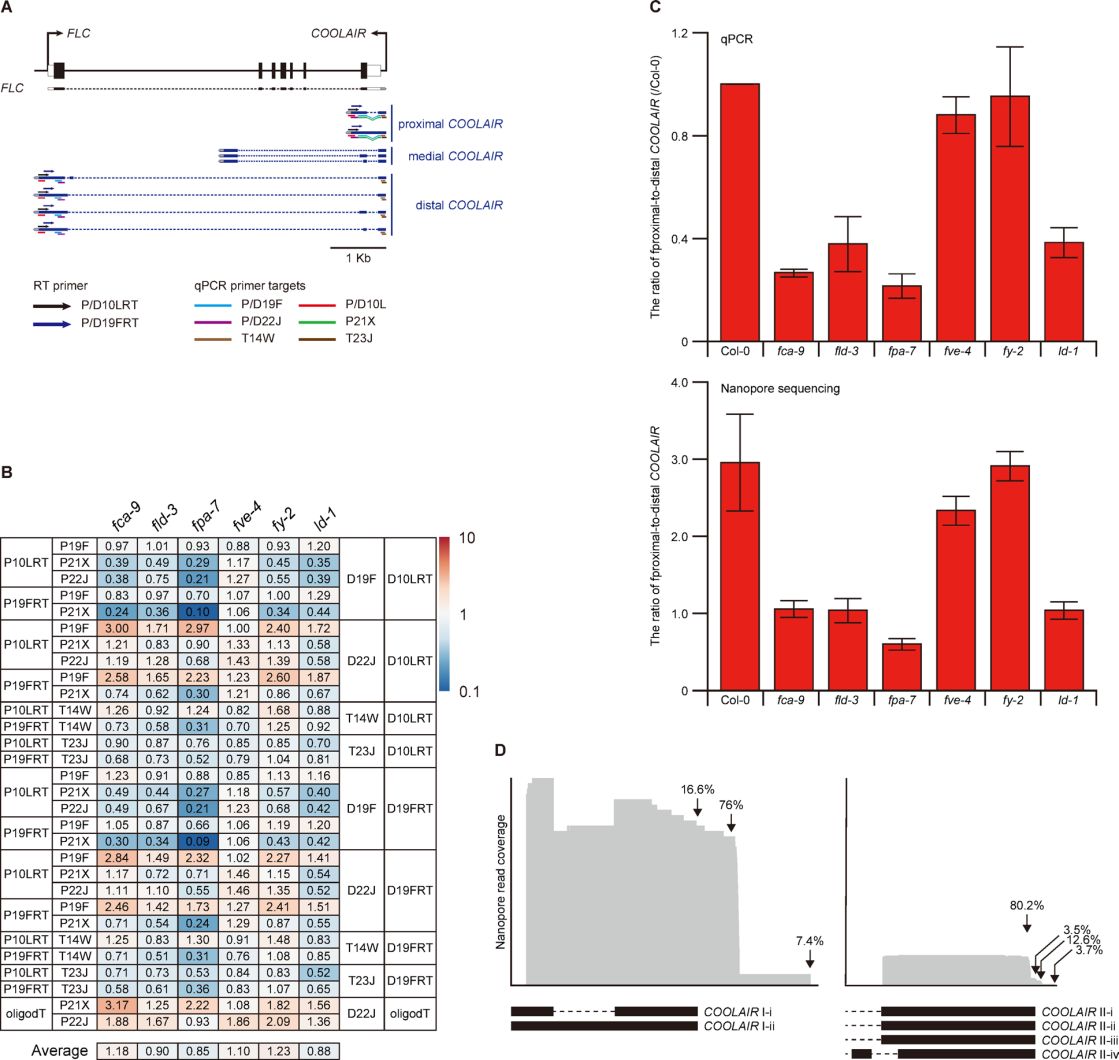
Application of STALARD to low-abundance *COOLAIR* isoforms. **A**. Schematic of the *FLC* locus, its transcripts, and primers used to calculate the proximal-to-distal *COOLAIR* ratio by qPCR. Colored boxes indicate exons (black for sense; blue for antisense), dashed lines indicate introns, white boxes represent untranslated regions (UTR), and gray arrows indicate polyA tails. **B**. Heatmap of proximal/distal *COOLAIR* ratios obtained using various primer sets in *fca-9*, *fld-3*, *fpa-7*, *fve-4*, *fy-2*, and *ld-1*, measured by conventional RT-qPCR. Fold changes are normalized to Col-0, and ratios were calculated as proximal fold change divided by distal fold change. (C) Proximal/distal *COOLAIR* ratios measured using STALARD by qPCR and nanopore sequencing. For nanopore data, ratios were calculated directly from transcript counts (proximal divided by distal *COOLAIR*). Data represent mean ± SEM from three biological replicates. (D) Nanopore sequencing read coverage across the *COOLAIR* locus in STALARD samples. PolyA site usage was calculated from mapped reads

To test STALARD’s sensitivity on challenging targets, we applied it to *COOLAIR*, a low-abundance antisense non-coding RNA transcribed from the *FLC* locus. *COOLAIR* undergoes complex AS and alternative polyadenylation, yielding at least 9 isoforms classified as proximal (2 isoforms), medial (3 isoforms), and distal (4 isoforms) (Fig. 3A). These isoforms are dynamically polyadenylated by AP proteins [16–26]. In addition, it has been proposed that the proximal polyadenylation and epigenetic modification are coupled by AP proteins to repress *FLC* expression; thus, the ratio of proximal-to-distal *COOLAIR* transcripts is key evidence to support this model [16–26].

However, *COOLAIR* transcript levels are extremely low (TPM < 0.1), and previous RT-qPCR studies have reported inconsistent results due to differences in primer design [16–26]. We tested multiple combinations of transcript-specific RT and qPCR primers targeting proximal, distal, and total *COOLAIR* isoforms. Results varied widely between primer sets, even within the same genotype (Fig. 3B, Supplemental Fig. 2), undermining confidence in the data. For example, the P19FRT-P21XqPCR combination for proximal *COOLAIR* results in extremely low proximal/distal ratios in AP mutants except *fve-4*, while other primer combinations, P10LRT-P19FqPCR, showed the opposite trend. Such inconsistencies highlighted the need for a more reliable method.

We applied STALARD to amplify *COOLAIR* transcripts from Col-0 and AP mutants, followed by both qPCR and nanopore sequencing. Results from both methods were consistent, revealing a decreased proximal/distal ratio in *fca*, *fld*, *fpa*, and *ld* mutants, but not in *fve* (Fig. 3C)-in line with prior studies [19, 20, 22, 23, 26]. In these four mutants, distal *COOLAIR* levels increased ~ 5-fold more than proximal *COOLAIR*. Interestingly, *fy* showed a ratio similar to Col-0, suggesting FY may not contribute to *COOLAIR* processing, despite its known interaction with FCA. Overall, these results demonstrate that STALARD enables reliable quantification of extremely low-abundance *COOLAIR* isoforms and preserves relative transcript ratios.

### Determining 3’-end structures using STALARD with nanopore sequencing

Given its similarity to 3′ RACE [44], we further tested whether STALARD combined with nanopore sequencing could accurately identify alternative polyadenylation sites. As expected, analysis of PolyA-trimmed reads revealed multiple PolyA sites in both proximal and distal *COOLAIR* isoforms (Fig. 3D). For proximal *COOLAIR*, three polyadenylation sites were identified: only 16.6% of reads terminated at the annotated site (ARAPORT11), while 76% were polyadenylated at a previously unannotated site, 116 bases downstream. A third site, located 479 bases downstream of the annotated site, accounted for 7.4% of reads. Similarly, distal *COOLAIR* displayed four distinct polyadenylation sites. Only 3.5% of reads terminated at the annotated site, while the majority (80.2%) ended 20 bases upstream. Two additional minor sites, located 16 and 80 bases downstream of the annotated site, contributed 12.6% and 3.7% of reads, respectively. These results demonstrate that STALARD can be used not only for quantification but also for precise 3′-end mapping, offering capabilities comparable to 3′ RACE with improved sensitivity and simplicity. While STALARD resembles 3′ RACE in function, it is limited to polyadenylated RNAs and cannot detect transcripts lacking polyA tails. Nonetheless, its simplicity and sensitivity make it a practical alternative for 3′-end analysis of known isoforms.

## Discussion

Robust quantification of low-abundance RNA isoforms remains a major challenge in molecular biology, particularly in plant systems where developmental cues and environmental responses often hinge on subtle shifts in transcript abundance or alternative splicing (AS) events. STALARD addresses this gap by providing a simple, accessible approach for selectively amplifying target transcripts without requiring specialized equipment, deep sequencing, or large RNA inputs.

The distinguishing feature of STALARD lies in its design: by incorporating a gene-specific sequence into the oligo(dT) primer and using a single primer for PCR, the method selectively enriches transcripts that are polyadenylated and share a defined 5′-end. While STALARD is not suited for discovering novel transcription start sites (TSSs), it can detect and quantify both known and novel isoforms that differ downstream, such as those arising from alternative splicing or polyadenylation, as long as they originate from the same 5′-end. This feature makes STALARD a powerful tool for studies seeking to uncover previously uncharacterized 3′ transcript diversity, especially when used in combination with long-read sequencing platforms.

This capability is particularly useful when quantifying isoforms with extremely low abundance or variable 3′-end processing, such as *COOLAIR*. Our results show that STALARD not only enables reliable quantification of these isoforms but also reveals alternative 3′ ends that were not captured by standard annotation. Thus, STALARD serves as both a sensitive quantification platform and an enrichment tool for rare isoforms, bridging the gap between PCR-based detection and transcriptome-wide discovery.

In the context of plant research, where transcript isoforms such as those of *FLM*,* MAF2*, and *COOLAIR* play regulatory roles in flowering time and stress adaptation, STALARD allows researchers to resolve biologically meaningful differences that are otherwise masked by the detection limits of conventional RT-qPCR. Our results demonstrate that STALARD can reproducibly quantify isoforms such as *ATX2* and *COOLAIR* that are undetectable by conventional qPCR due to low Cq values near the LLOQ.

STALARD offers several advantages over alternative methods. Compared to digital PCR (dPCR), which also improves detection sensitivity but requires specialized reagents and instruments, STALARD can be performed with standard laboratory tools. While RNA sequencing (RNA-seq) provides transcriptome-wide coverage, it is often inefficient for accurately quantifying low-abundance isoforms without deep sequencing and complex bioinformatics. STALARD provides a targeted, cost-effective alternative with high sensitivity and reproducibility.

However, as with any method, STALARD has limitations. Because it relies on a gene-specific primer corresponding to the known 5′ end of the target transcript, isoforms with alternative transcription start sites or truncated 5′ ends will not be amplified. STALARD is therefore not intended for full isoform discovery, as it cannot detect transcripts with uncharacterized 5′ ends. Additionally, its current implementation depends on oligo(dT)-based reverse transcription, restricting its application to polyadenylated transcripts. Future adaptations using alternative priming strategies or template-switching technologies may broaden its applicability to non-polyadenylated RNAs.

Another practical consideration is PCR cycle number. Amplification beyond 12 cycles may introduce non-specific products, particularly in qPCR applications. While STALARD is robust under standard conditions, careful optimization of primer design and cycle number is recommended for new or complex transcript targets.

In summary, STALARD offers a practical solution to a longstanding bottleneck in transcript quantification. It enables sensitive, isoform-specific analysis of low-abundance polyadenylated RNAs using standard reagents and instrumentation. While it requires prior knowledge of the 5′ end sequence, this design also confers its specificity and enrichment power. Its simplicity, cost-effectiveness, and compatibility with both qPCR and sequencing platforms make it a valuable addition to the molecular toolkit for transcript-level studies in plants and beyond.

## Conclusions

STALARD is a streamlined, cost-effective method for amplifying and quantifying low-abundance RNA isoforms using standard reverse transcription and PCR reagents. It preserves relative isoform abundance and enables reliable quantification even for transcripts near or below the qPCR detection limit. We demonstrated its effectiveness across a range of targets, including *VIN3*,* FLM*,* MAF2*,* EIN4*,* ATX2*, and *COOLAIR*, using both qPCR and nanopore sequencing. STALARD also facilitates precise 3′-end mapping, as shown by the identification of previously unannotated polyadenylation sites in *COOLAIR* isoforms. Importantly, STALARD can also detect novel isoforms that differ in their 3′ structures, provided they originate from a defined 5′-end and are polyadenylated. Overall, STALARD offers a powerful, accessible approach for isoform-specific RNA analysis, especially for low-abundance and non-coding transcripts.

## Supplementary Information

Below is the link to the electronic supplementary material.

Supplementary Material 1.

Supplementary Material 2.

## Data Availability

No datasets were generated or analysed during the current study.

## Abbreviations

AP: Autonomous pathway
AS: Alternative splicing
ATX2: ARABIDOPSIS TRITHORAX2
Cq: Quantification cycles
dPCR: Digital PCR
EIN4: ETHYLENE INSENSITIVE4
FLC: FLOWERING LOCUS C
FLM: FLOWERING LOCUS M
FRI: FRIGIDA
GSoligo(dT): GSP-tailed oligo(dT)_24_VN
GSP: Gene-specific primer
LD: Long-day
LLOQ: Lower limit of quantification
LOD: Limit of detection
MAF2: MADS AFFECTING FLOWERING2
MIQE: Minimum information for publication of quantitative Real-time PCR experiments
mRNA: Messenger RNA
MS: Murashige and Skoog
NV: Non-vernalized
qPCR: Quantitative real-time polymerase chain reaction
RACE: Rapid amplification of cDNA ends
RT-qPCR: Reverse transcription-quantitative real-time polymerase chain reaction
SD: Short-day
SMART: Switching mechanism at 5’-end of RNA template
STALARD: Selective target amplification for low-abundance RNA detection
Tm: Melting temperature
TPM: Transcripts per million
UBC21: UBIQUITIN-CONJUGATING ENZYME21
VIN3: VERNALIZATION INSENSITIVE3
