# Supplementary material for: STALARD: Selective Target Amplification for Low-Abundance RNA Detection
Source: Plant Methods. 2025 Sep 29;21:123. doi: 10.1186/s13007-025-01443-z (PMC12482509; doi:10.1186/s13007-025-01443-z)
Supplement: Supplementary file 1 — Supplementary Material 1. [file 13007_2025_1443_MOESM1_ESM.docx]

**Supplementary Figures**

**Supplementary Figure 1**


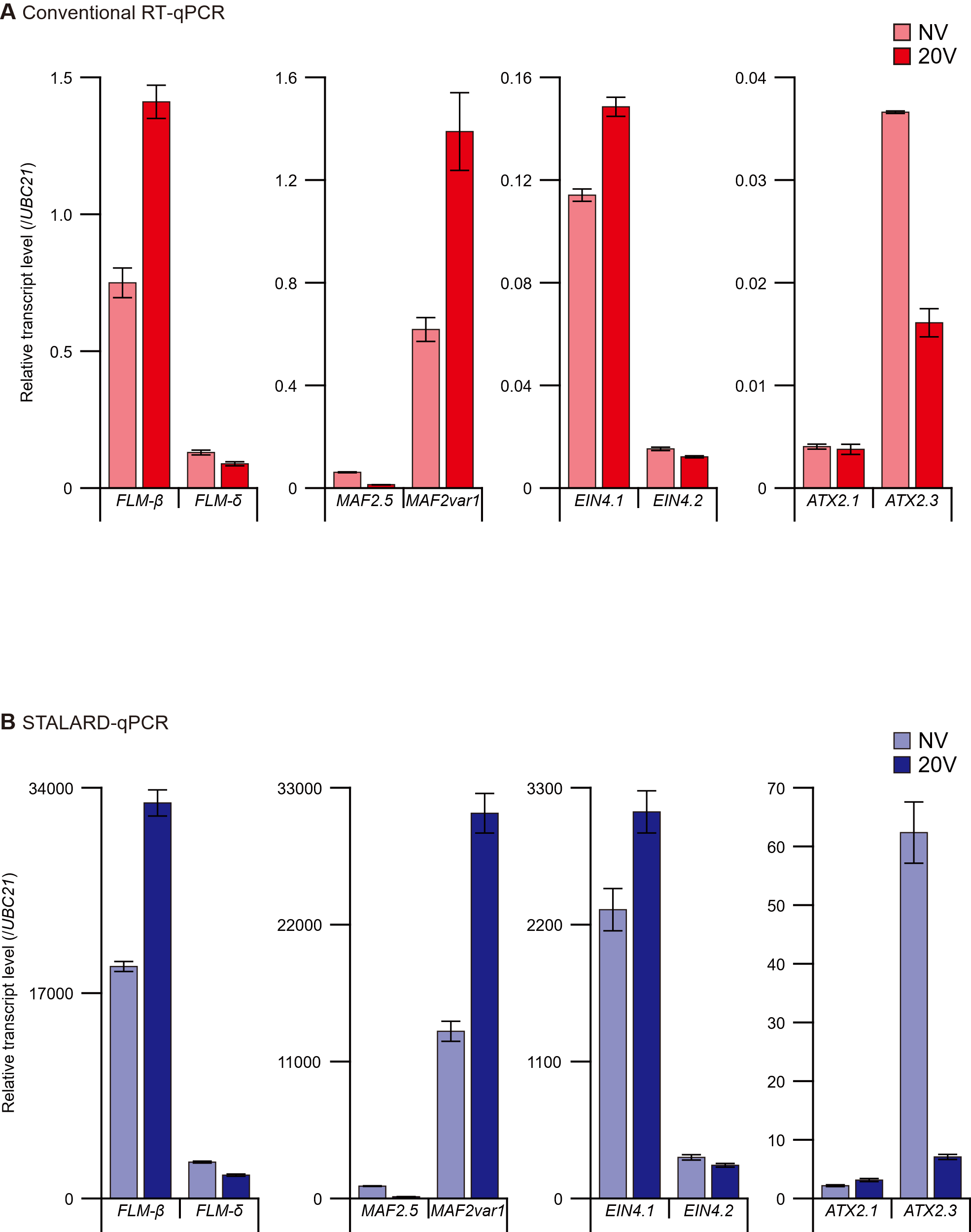


**Supplementary Figure 1.** Relative transcript levels of alternatively spliced isoforms.

**A**. Relative transcript levels of alternatively spliced isoforms under NV and 20V conditions, measured by conventional RT-qPCR. Data represent the mean ± SEM of three biological replicates. **B**. Relative transcript levels under NV and 20V conditions measured by STALARD-qPCR. Data represent the mean ± SEM from three biological replicates.

**Supplementary Figure 2**


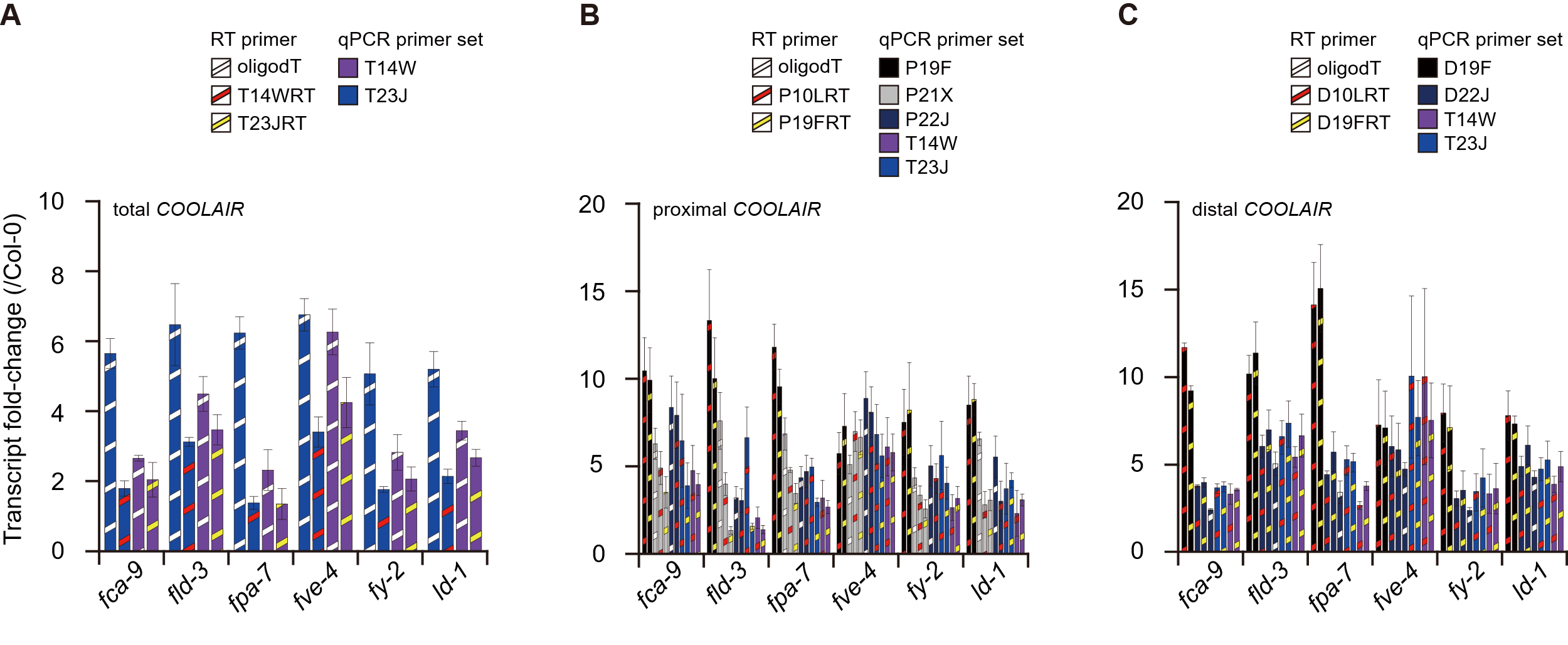


**Supplementary Figure 2.** Fold changes of *COOLAIR* transcript levels.

Fold changes in total *COOLAIR* (**A**), proximal *COOLAIR* (**B**), and distal *COOLAIR* (**C**) transcript levels in AP mutants relative to Col-0, quantified by conventional RT-qPCR using various primer sets. Data represent the mean ± SEM of three biological replicates.
